# SHANK3 Downregulation in the Ventral Tegmental Area Accelerates the Extinction of Contextual Associations Induced by Juvenile Non-familiar Conspecific Interaction

**DOI:** 10.3389/fnmol.2018.00360

**Published:** 2018-10-11

**Authors:** Sebastiano Bariselli, Alessandro Contestabile, Stamatina Tzanoulinou, Stefano Musardo, Camilla Bellone

**Affiliations:** Department of Fundamental Neuroscience, University of Geneva, Centre Médical Universitaire (CMU), Geneva, Switzerland

**Keywords:** conditioned place preference, social novelty, VTA, Shank3, isolation

## Abstract

Haploinsufficiency of the *SHANK3* gene, encoding for a scaffolding protein located in the postsynaptic density of glutamatergic synapse, has been linked to forms of autism spectrum disorders (ASDs). It has been shown that SHANK3 controls the maturation of social reward circuits in the ventral tegmental area (VTA). Whether the impairments in associative learning observed in ASD relate to SHANK3 insufficiency restricted to the reward system is still an open question. Here, we first characterize a social-conditioned place preference (CPP) paradigm based on the direct and free interaction with a juvenile and non-familiar conspecific. In both group- and single-housed C57Bl6/j late adolescence male mice, this CPP protocol promotes the formation of social-induced contextual associations that undergo extinction. Interestingly, the downregulation of *Shank3* expression in the VTA altered the habituation to a non-familiar conspecific during conditioning and accelerated the extinction of social-induced conditioned responses. Thus, inspired by the literature on drugs of abuse-induced contextual learning, we propose that acquisition and extinction of CPP might be used as behavioral assays to assess social-induced contextual association and “social-seeking” dysfunctions in animal models of psychiatric disorders.

## Introduction

Haploinsufficiency of the *SHANK3* gene, which encodes for a scaffolding protein located in the postsynaptic density of glutamatergic synapses, has been causally linked to Phelan McDermid syndrome. This syndrome is characterized by intellectual disability, developmental delay, poor motor function and autism spectrum disorder (ASD). To find possible therapeutic interventions, it is fundamental to identify the causative mechanisms and the main neuronal circuits involved in the disease. In humans, emerging evidence suggests an existing association between reward system alterations and ASD (Chevallier et al., 2012). Furthermore, it has been observed that infants with ASD have a diminished response to social reward stimuli that might relate to impairments in social learning (Scott-Van Zeeland et al., 2010). Although *Shank3* knock-out (KO) mice displayed deficits in reinforcement learning (Wang et al., 2016), the contribution of reward system dysfunctions to aberrant behavioral traits observed in ASD animal models remains largely unknown.

SHANK3 is a postsynaptic protein expressed at excitatory inputs where it controls transmission and plasticity (Naisbitt et al., 1999). In mice, the *Shank3* gene is composed of 21 exons with several internal promoters (Jiang and Ehlers, 2013), which encodes for a variety of protein isoforms. Considering the association between ASD and *SHANK3* mutations in humans, several *Shank3* mutant mouse lines have been generated to study both the contribution of the gene to the emergence of ASD-related symptoms and the neuronal circuit dysfunctions underlying aberrant behavioral traits. Sociability, social preference and social novelty recognition abnormalities have been observed with variable severity in mutant mice lacking the different SHANK3 isoforms (Ferhat et al., 2017). Besides its role in controlling sociability and stereotypes in mice, the expression of certain SHANK3 variants is necessary for appropriate learning. In fact, both exon 4–9 and exon 21 deletion lead to impairment in spatial learning (Kouser et al., 2013) and, depending on the genetic background, exon 4–9 KO showed alterations in fear conditioning (Wang et al., 2011; Yang et al., 2012; Drapeau et al., 2014). Recently, it has been demonstrated that a full KO mouse line for all SHANK3 isoforms displays deficits in reinforcement learning (Wang et al., 2016). However, whether the impairments in associative learning are attributable to a SHANK3 insufficiency restricted to the reward system is still an open question.

Rodents can learn to associate environmental cues (conditioned stimuli, CS) with the availability of positive experiences, such as social interaction (unconditioned stimuli, US) resulting in conditioned place preference (CPP) responses. CPP tasks, classically used to study the reinforcing properties of drugs of abuse (Tzschentke, 2007), have been adopted to test the ability of conspecific interaction to promote contextual associative learning (Calcagnetti and Schechter, 1992; Crowder and Hutto, 1992; Van den Berg et al., 1999; Panksepp and Lahvis, 2007; Thiel et al., 2008; Trezza et al., 2009; Kummer et al., 2011; Dölen et al., 2013; Lahvis et al., 2015; Hung et al., 2017). Contextual associations are subject to extinction, a behavioral phenomenon caused by the repeated presentations of contextual cues in the absence of the reinforcer and characterized by a timely reduction of the conditioned responses (for a comprehensive view on extinction theories see Bouton, 2004; Quirk and Mueller, 2008; Dunsmoor et al., 2015). However, although it has been shown that social-induced CPP undergoes extinction in adolescent rats (Trezza et al., 2009), the involvement of SHANK3 in this phenomenon remains elusive.

The reward system originates from the highly heterogeneous dopamine (DA) neurons of the ventral tegmental area (VTA) and controls reinforcement learning. Despite their diversity, at least a subclass of midbrain DA neurons increase their activity in response to unexpected rewards and, after associative learning, to cues predicting reward availability (Schultz et al., 1997; Cohen et al., 2012). Importantly, while optogenetic studies demonstrated the sufficiency of VTA DA neuron stimulation to promote learning (Tsai et al., 2009; Adamantidis et al., 2011), blockade of DA receptors confirmed the necessity of intact DA signaling for reward-induced place preference acquisition (Hoffman and Beninger, 1989; Cunningham et al., 2000; Rezayof et al., 2002; Le Foll et al., 2005; Vidal-Infer et al., 2012). Appropriate synaptic transmission and plasticity at synaptic inputs onto VTA DA neurons are fundamental, not only for the acquisition (Stuber et al., 2008; Huang et al., 2016) but also for the extinction of reinforcement learning (Engblom et al., 2008). Emerging evidence suggests the involvement of the DA system in social-induced learning of new contextual associations. In fact, social-induced CPP activates several regions within the reward system (El Rawas et al., 2012), it is controlled by oxytocin within the VTA and NAc (Dölen et al., 2013; Hung et al., 2017) and is impaired by the DA re-uptake inhibitor, methylphenidate (Trezza et al., 2009). Recently, we demonstrated that an intact excitability and expression of the ASD-associated protein Neuroligin 3 at VTA DA neurons (Bariselli et al., 2018) are necessary for the acquisition of social-induced CPP. However, the role of SHANK3 at VTA neurons in learning and extinction of social-induced contextual associations remained unexplored.

To investigate the involvement of SHANK3 at VTA neurons in controlling social-induced associative learning, we first characterized a social CPP paradigm (Bariselli et al., 2018) based on the direct and free interaction with a juvenile and non-familiar conspecific. The acquisition of CPP does not rely on single-housing and undergoes extinction. Moreover, we found that while the downregulation of *Shank3* expression in the VTA did not prevent the acquisition of a preference for the non-familiar conspecific-associated compartment, it altered the habituation to non-familiar conspecific and accelerated the extinction of social-induced conditioned responses.

## Materials and Methods

### Animals

The experimental procedures described here were conducted in accordance with the Swiss laws and previously approved by the Geneva Cantonal Veterinary Authority. Male C57Bl/6j mice were purchased from Charles River Laboratories and housed in the institutional animal facility under standard 12 h/12 h light/dark cycles with food and water *ad libitum*. Experimental animals were group-housed (2–3 per cage), or single-housed only for the single-housing experimental condition, and tested during late adolescence, at the 8th or 9th week of life. Younger non-familiar male mice (3–4 weeks; sex-matched) were single-housed and used as stimuli animals during the conditioning sessions of the CPP protocol. Behavioral experiments were conducted in a room with fixed illumination (20 Lux) at a temperature between 22°C and 24°C. The experiments were performed in a time window that started approximately 2 h after the end of the dark circle and ended 2 h before the start of the next dark circle.

### Real-Time Quantitative Reverse Transcriptase PCR

Total RNA was extracted using the RNeasy PLUS Mini Kit (Qiagen), from the VTA of mice infected either with shSHANK3 or scrSHANK3. After quantification with Nanodrop Spectrophotometer, 1 μg of RNA was retrotranscribed in cDNA with QuantiTect Reverse Transcription Kit (Qiagen), followed by a PCR amplification of the subsequent transcripts: *SHANK3* forward primer 5′ acgaagtgcctgcgtctggac 3′, reverse primer 5′ ctcttgccaaccattctcatcagtg 3′, *Tyrosine hydroxylase (TH)* forward primer 5′ ccccacctggagtattttgtg 3′, reverse primer 5′ atcacgggcggacagtagacc 3′, *Actin* forward primer 5′ agagggaaatcgtgcgtgac 3′, reverse primer 5′ caatagtgatgacctggccgt 3′. Reactions were carried out using iTaq™ Universal SYBR^®^ Green Supermix (Biorad) on Applied Biosystems 7,500 Real-Time PCR System (Applied Biosystems, Foster City, CA, USA) by 50°C for 2 min, 95°C for 10 min followed by 40 cycles at 95°C for 15 s and 60°C for 1 min. Melting curve analyses were performed to verify the amplification specificity. Relative quantification of gene expression was performed according to the ΔΔ-Ct method (Livak and Schmittgen, 2001).

### Conditioned Place Preference Induced by Non-familiar Conspecific Interaction

**Figure 1:**
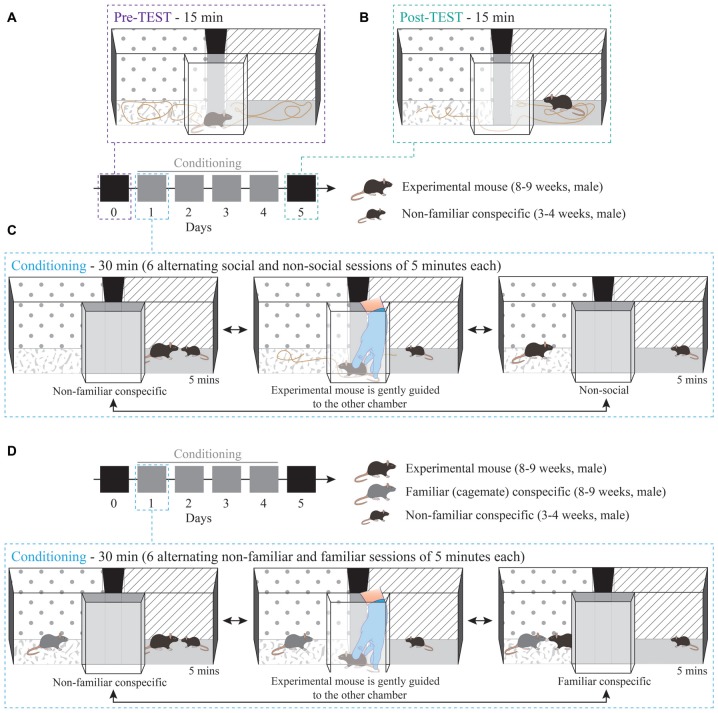
Non-familiar conspecific-induced conditioned place preference (CPP): a variation of social-induced CPP to assess the reinforcing properties of social interaction. Schematic 3D representation of the CPP procedure and apparatus. The protocol consisted of: **(A)** 15 min pre-TEST, **(B)** 15 min post-TEST and **(C)** conditioning phase (30 min per day for four consecutive days). **(D)** Schematic 3D representation of conditioning phase for non-familiar vs. familiar social stimuli protocol, during which the experimental mouse interacted with a non-familiar social stimulus in one chamber and with a familiar (cage-mate) stimulus in the other one.

The apparatus (Bioseb, Model BIOSEB, *in vivo* Research Instruments, spatial place preference box for mice LE895) used for the CPP protocol consists of two square-shaped chambers (20 × 20 cm) with either gray stripes on white background or black dots on white background. The floor in each of the two chambers has different distinct textures, namely one with a smooth and one with a rough surface. The two chambers are interconnected by a small corridor, with transparent walls and floor, and removable doors allow the corridor to be closed (Figure 1).

After each pre-TEST, conditioning session, post-TEST and extinction protocol the arena was cleaned thoroughly with 1% acetic acid and allowed to dry before continuing with the next animal.

*Day 0—Pre-TEST*: experimental mice freely explored the CPP apparatus for 15 min to establish a baseline preference for both chambers (Figure 1A). On the same day, stimuli mice were habituated either to one or the other chamber for 15 min. The chamber that they were habituated in was the chamber that they were in during the conditioning sessions.

*Days 1–4—Conditioning, paired protocol (non-familiar vs. non-social session)*: for each experimental mouse, one chamber was randomly assigned as the paired session chamber, with the presence of a non-familiar mouse (US+) and the other as the non-social session chamber (US). This protocol remained stable for each mouse throughout the conditioning sessions of Days 1–4. Experimental mice were subjected to six alternating conditioning sessions of 5 min each, resulting in 30 min of total conditioning time per day (e.g., non-familiar mouse session 1, non-social session 1, non-familiar mouse session 2, non-social session 2, non-familiar mouse session 3, non-social session 3; Figure 1C). During each conditioning session, the experimental subjects were allowed to freely interact with social stimuli. To counteract bias formation, each day the starting session was counterbalanced across mice and alternated for each mouse, such that, if for example on day 1 the experimental subject was exposed to the paired session first, on day 2 the conditioning started with the non-social session. After the end of each 5-min trial, the animals were gently guided by the experimenter through the corridor and towards the other chamber (Figure 1C), therefore the animals were exposed to the spatial orientation of both chambers and apparatus.

*Days 1–4—Conditioning, contingency break protocol*: this experimental protocol did not maintain the stable contingencies between chamber and stimulus. Specifically, the assignment of one context to the non-familiar stimulus (US+) and another context to the non-social (US) was inverted each day (e.g., day 1: non-familiar mouse in chamber A vs. non-social in chamber B; day 2: non-familiar mouse in chamber B vs. non-social in chamber A; day 3: non-familiar mouse in chamber A vs. non-social in chamber B; and day 4: non-familiar mouse in chamber B vs. non-social in chamber A).

*Days 1–4—Conditioning, paired protocol (familiar session vs. non-familiar session)*: for each experimental mouse one chamber was assigned as the paired session chamber, with the presence of the non-familiar mouse (US+), and the other chamber was associated with the presence of a cage-mate, the familiar stimulus (US). This protocol remained stable for each mouse throughout the conditioning sessions of Days 1–4. Experimental mice were subjected to six alternating conditioning sessions of 5 min each, resulting in 30 min of total conditioning time per day (e.g., non-familiar mouse session 1, familiar mouse session 1, non-familiar mouse session 2, familiar mouse session 2, non-familiar mouse session 3, familiar mouse session 3; Figure 1D). Each day the starting session was counterbalanced across mice and alternated for each mouse. After the end of each 5-min trial, the animals were gently guided by the experimenter through the corridor and towards the other chamber (Figure 1D).

We should note that, in this paradigm, the experimental mouse was free to interact with a social stimulus that remained unchanged throughout all the conditioning sessions. Typically, one stimulus mouse was assigned to one experimental animal. Thus, although the stimulus mouse represented a stranger stimulus at the first conditioning session, the non-familiarity aspect was maintained since both experimental and stimuli animals returned to their respective home-cages and kept separated after each conditioning day. Thus, in this case, the US exposure represented a valued condition. In contrast, when the experimental subject had access to the familiar stimulus both in the home-cage and in the conditioned side of the apparatus, the familiar stimulus mouse exposure represented a devalued condition.

*Day 5—Post-TEST*: 24 h after the last conditioning session, experimental mice were placed in the corridor of the CPP apparatus and, after lifting the removable doors, the animals could freely explore the arena once again for 15 min and establish a preference (Figure 1B) in the absence of the US.

*Extinction*: starting 48 h after the post-TEST session, experimental mice were exposed for several days (5–6 days) to the empty apparatus. In particular, mice were placed in the corridor of the CPP apparatus and, after lifting the removable doors, explored the arena for 15-min-long sessions in the absence of the US. After each extinction session, the arena was cleaned thoroughly with 1% acetic acid and allowed to dry before continuing with the next animal.

The behavior of the animals was tracked automatically with the Ethovision XT software (Noldus, Wageningen, Netherlands) or AnyMAZE and the time spent in the two chambers was recorded for the pre- and post-TEST sessions. Subsequently, the preference index was calculated as: time spent in the non-familiar conspecific-paired chamber divided by the time spent in the non-social chamber for *non-familiar vs. non-social session*, and time spent in the non-familiar conspecific-paired chamber divided by the time spent in the familiar-paired chamber for *familiar session vs. non-familiar session*. Moreover, we calculated the learning index by dividing the preference index calculated at post-TEST by the preference index calculated at pre-TEST. For all behavioral cohorts, the parameters of body and nose-to-nose contact, number of transitions and length of mean visit were analyzed using Ethovision XT.

### Stereotaxic Injections

Purified scrShank3 and shShank3 (AAV1-GFP-U6-scrmb-shRNA; titer: 5.9 × 10^13^ GC/mL and AAV5-ZacF-U6-luc-zsGreen-shShank3; titer: 7.4 × 10^13^ GC/mL, VectorBioLab) injections were delivered in mice younger than P6 as previously described (Bariselli et al., 2016). After anesthesia induction with a mixture of isoflurane/O_2_, C57Bl/6j wildtype pups were placed on a stereotaxic frame (Angle One; Leica, Germany) and a single craniotomy was made over the VTA. To obtain bilateral VTA infection, 200 nl of viral solution was injected at the following coordinates: ML: 0.15 mm, AP: 0.1 mm, DV: −4.0 and −3.9 mm from lambda through a graduated glass pipette (Drummond Scientific Company, Broomall, PA, USA). After behavioral experiments, *post hoc* analysis was performed to validate the localization of the infection (Figure 6B).

**Figure 2:**
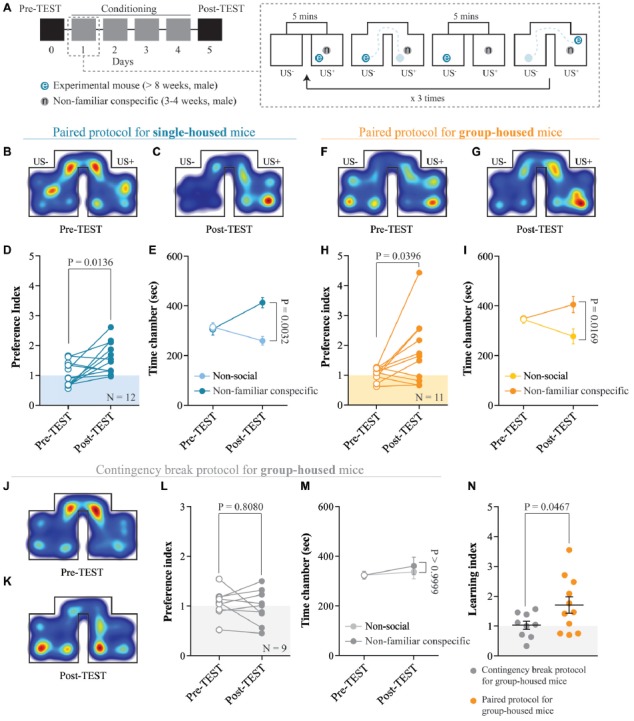
Acquisition of non-familiar conspecific-induced CPP does not require single-housing. **(A)** Left: schematic representation of CPP time-course. Right: schematic representation of a single CPP conditioning alternation. **(B)** Representative apparatus occupancy heat-maps for single-housed mice during pre-TEST and **(C)** post-TEST. **(D)** Preference index calculated at pre- and post-TEST for single-housed mice (*N* = 12; paired *t*-test: *t*_(11)_ = 2.934; *P* = 0.0136). **(E)** Time spent in non-social and non-familiar conspecific-paired chambers for single-housed mice during pre- and post-TEST (*N* = 12; two-way RM ANOVA: time × chamber interaction: *F*_(1,11)_ = 4.021, *P* = 0.0702; main effect chamber: *F*_(1,11)_ = 9.85, *P* = 0.0094; main effect time: *F*_(1,11)_ = 6.363, *P* = 0.0284; followed by Bonferroni’s multiple comparisons test). **(F)** Representative apparatus occupancy heat-maps for group-housed animals at pre-TEST and **(G)** post-TEST. **(H)** Preference index calculated at pre- and post-TEST for group-housed mice (*N* = 11; paired *t*-test: *t*_(10)_ = 2.366; *P* = 0.0396). **(I)** Time spent in the non-social and non-familiar conspecific-associated chambers for group-housed mice during pre- and post-TEST (*N* = 11; two-way RM ANOVA: time × chamber interaction: *F*_(1,10)_ = 5.115, *P* = 0.0472; main effect chamber: *F*_(1,10)_ = 3.019, *P* = 0.1129; main effect time: *F*_(1,10)_ = 0.2936, *P* = 0.5998; followed by Bonferroni’s multiple comparisons test). **(J)** Representative apparatus occupancy heat-maps of group-housed mice subject to CPP contingency break at pre-TEST and **(K)** post-TEST. **(L)** Preference index of group-housed mice subjected to CPP contingency break calculated at pre- and post-TEST (*N* = 9; paired *t*-test: *t*_(8)_ = 0.6537; *P* = 0.8080). **(M)** Time spent in the two chambers of the apparatus for group-housed mice subjected to CPP contingency break during pre- and post-TEST (the chambers were assigned a non-social and non-familiar property for analyses purposes; *N* = 9; two-way RM ANOVA: time × chamber interaction: *F*_(1,8)_ = 0.1864, *P* = 0.6773; main effect chamber: *F*_(1,8)_ = 0.1024, *P* = 0.7572; main effect time: *F*_(1,8)_ = 2.401, *P* = 0.1599; followed by Bonferroni’s multiple comparisons test). **(N)** Learning index calculated for group-housed mice subjected to either a contingency break (*N* = 9) or a paired protocol CPP (*N* = 11; unpaired *t*-test with Welch’s correction: *t*_(14.4)_ = 2.175; *P* = 0.0467). *N* indicates number of mice. Abbreviations: US, unconditioned stimulus.

**Figure 3:**
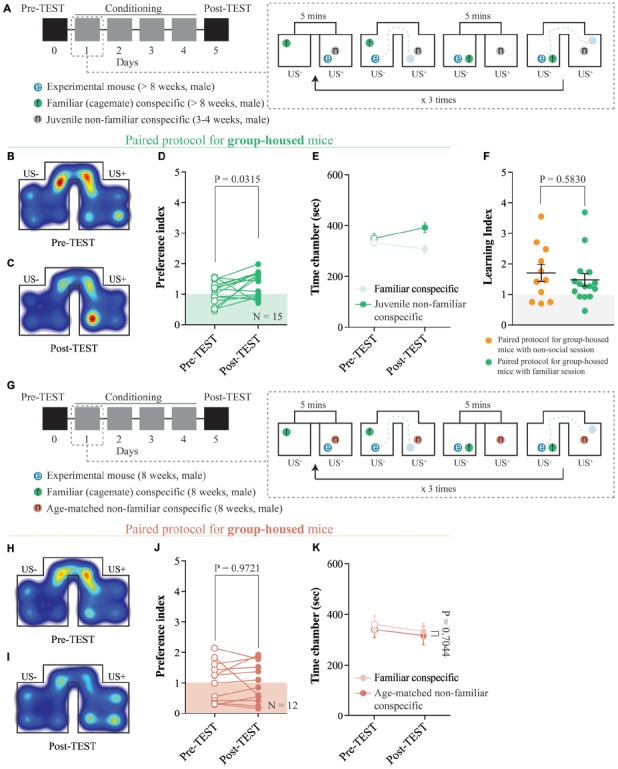
The interaction with a juvenile non-familiar conspecific is reinforcing. **(A)** Left: schematic representation of CPP time-course. Right: schematic representation of a single CPP conditioning alternation between a familiar session and a non-familiar conspecific session. **(B)** Representative apparatus occupancy heat-maps during pre-TEST and **(C)** post-TEST for group-housed mice subjected to this protocol. **(D)** Preference index calculated at pre- and post-TEST for group-housed mice subjected to this protocol (*N* = 15; paired *t*-test: *t*_(14)_ = 2.389; *P* = 0.0315). **(E)** Time spent in familiar and non-familiar conspecific-paired chambers for group-housed mice subjected to this protocol during pre- and post-TEST (*N* = 15; two-way RM ANOVA: time × chamber interaction: *F*_(1,14)_ = 4.525, *P* = 0.0517; main effect chamber: *F*_(1,14)_ = 3.416, *P* = 0.0858; main effect time: *F*_(1,14)_ = 1.657, *P* = 0.2189). **(F)** Learning index calculated for group-housed mice subjected to either the protocol reported in Figure 2A (*N* = 11) or the protocol reported here in **(A)** (*N* = 15; Mann-Whitney test; *P* = 0.5830). **(G)** Left: schematic representation of CPP time-course. Right: schematic representation of a single CPP conditioning alternation between a familiar session and a non-familiar conspecific session. **(H)** Representative apparatus occupancy heat-maps during pre-TEST and **(I)** post-TEST for group-housed mice subjected to this protocol. **(J)** Preference index calculated at pre- and post-TEST for group-housed mice subjected to this protocol (*N* = 12; paired *t*-test: *t*_(11)_ = 0.0358; *P* = 0.9721). **(K)** Time spent in familiar and non-familiar conspecific-paired chambers for group-housed mice subjected to this protocol during pre- and post-TEST (*N* = 12; two-way RM ANOVA: time × chamber interaction: *F*_(1,11)_ = 0.0157, *P* = 0.9026; main effect chamber: *F*_(1,11)_ = 0.0927, *P* = 0.7665; main effect time: *F*_(1,11)_ = 11.1, *P* = 0.0067). *N* indicates number of mice. Abbreviations: US, unconditioned stimulus.

**Figure 4:**
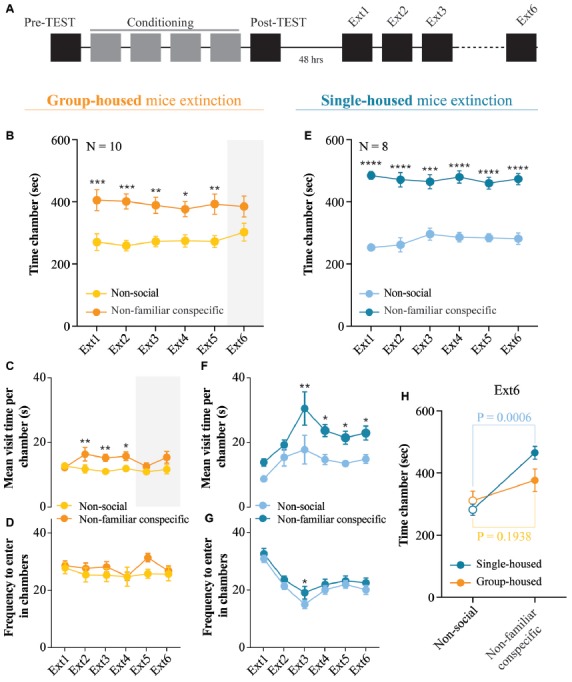
Extinction of non-familiar conspecific-induced CPP is affected by housing condition. **(A)** Schematic representation of CPP acquisition and extinction. **(B)** Time spent in non-social and non-familiar conspecific-paired chambers across extinction sessions for group-housed mice subjected to CPP (*N* = 10; two-way RM ANOVA: time × chamber interaction: *F*_(5,45)_ = 0.4486, *P* = 0.8120; main effect chamber: *F*_(1,9)_ = 8.484, *P* = 0.0172; main effect time: *F*_(5,45)_ = 1.25, *P* = 0.3021; followed by Bonferroni’s multiple comparisons test). **(C)** Mean visit time spent in non-social and non-familiar conspecific-paired chambers across extinction sessions for group-housed mice subjected to CPP (*N* = 10; two-way RM ANOVA: time × chamber interaction: *F*_(5,45)_ = 2.642, *P* = 0.0354; main effect chamber: *F*_(1,9)_ = 9.002, *P* = 0.0149; main effect time: *F*_(5,45)_ = 1.589, *P* = 0.1827; followed by Bonferroni’s multiple comparisons test). **(D)** Frequency to enter in non-social and non-familiar conspecific-paired chambers across extinction sessions for group-housed mice subjected to CPP (*N* = 10; two-way RM ANOVA: time × chamber interaction: *F*_(5,45)_ = 1.215, *P* = 0.3175; main effect chamber: *F*_(1,9)_ = 2.086, *P* = 0.1826; main effect time: *F*_(5,45)_ = 1.361, *P* = 0.2568). **(E)** Time spent in non-social and non-familiar conspecific-paired chambers across extinction sessions for single-housed mice (*N* = 8) subjected to a paired protocol (two-way RM ANOVA: time × chamber interaction: *F*_(5,35)_ = 1.037, *P* = 0.4113; main effect chamber: *F*_(1,7)_ = 78.38, *P* < 0.0001; main effect time: *F*_(5,35)_ = 0.997, *P* = 0.4339; followed by Bonferroni’s multiple comparisons test). **(F)** Mean visit time spent in non-social and non-familiar conspecific-paired chambers across extinction sessions for single-housed mice (*N* = 8) subjected to a paired protocol (two-way RM ANOVA: time × chamber interaction: *F*_(5,35)_ = 0.6993, *P* = 0.6268; main effect chamber: *F*_(1,7)_ = 11.16, *P* = 0.0086; main effect time: *F*_(5,35)_ = 9.897, *P* < 0.0001; followed by Bonferroni’s multiple comparisons test). **(G)** Frequency to enter in non-social and non-familiar conspecific-paired chambers across extinction sessions for single-housed mice (*N* = 8) subjected to a paired protocol (two-way RM ANOVA: time × chamber interaction: *F*_(5,35)_ = 0.6539, *P* = 0.6600; main effect chamber: *F*_(1,7)_ = 5.746, *P* = 0.0401; main effect time: *F*_(5,35)_ = 18.08, *P* < 0.0001; followed by Bonferroni’s multiple comparisons test). **(H)** Time spent in non-social or non-familiar conspecific-paired chamber at extinction session 6 for single-housed (*N* = 8) and group-housed (*N* = 10) mice (two-way ANOVA: time × group: *F*_(1, 32)_ = 3.316, *P* = 0.0780; main effect group: *F*_(1, 32)_ = 1.149, *P* = 0.2917; main effect time: *F*_(1, 32)_ = 23.09, *P* < 0.0001; followed by Bonferroni’s multiple comparisons test). *N* indicates number of mice. Significance: *p < 0.05, **p < 0.01, ***p < 0.001, ****p < 0.0001.

**Figure 5:**
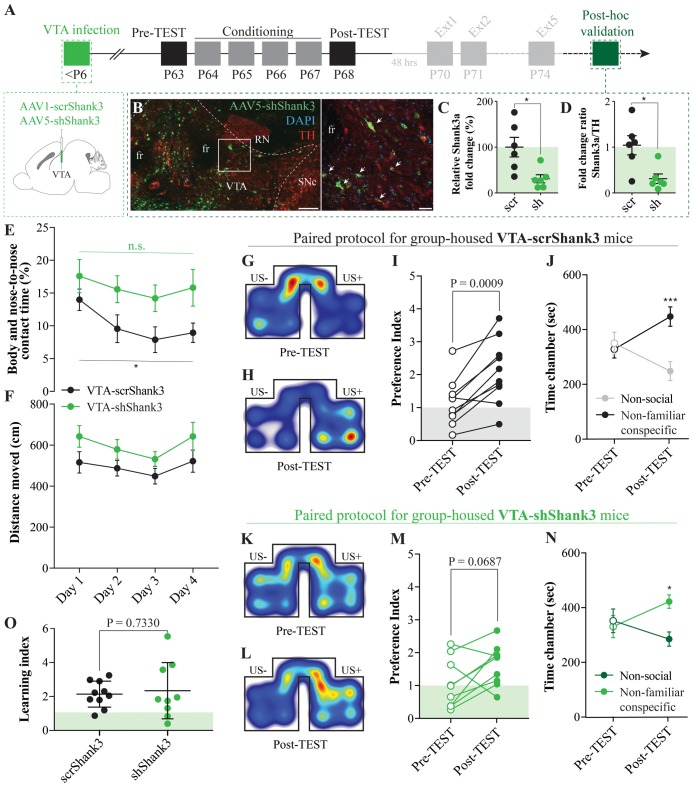
Ventral tegmental area (VTA) SHANK3 downregulation alters the exploration of non-familiar conspecific without affecting CPP. **(A)** Experimental time course of AAV-scrShank3/AAV-shShank3 VTA infection, CPP acquisition, extinction and *post hoc* histological verification of viral infection and effect. **(B)** Histological validation of VTA infection (scale bar: left panel: 150 μm, right panel: 50 μm; white arrows indicate tyrosine hydroxylase (TH)-shShank3 colocalization). **(C)** Relative expression of Shank3a after infection of AAV-scrShank3/AAV-shShank3 in the VTA (unpaired *t*-test: *t*_(10)_ = 2.972; *P* = 0.0140). **(D)** Ratio between Shank3a and TH expression after AAV-scrShank3/AAV-shShank3 infection in the VTA (Mann-Whitney test: *P* = 0.0152). **(E)** Body and nose-to-nose contact time during conditioning days for group-housed VTA-scrShank3 mice (*N* = 10) and VTA-shShank3 (*N* = 9) mice (two-way ANOVA: time × group interaction: *F*_(3,51)_ = 0.4552, *P* = 0.7148; group main effect: *F*_(1,17)_ = 4.894, *P* = 0.0409; main effect time: *F*_(3,51)_ = 4.363, *P* = 0.0082; followed by Bonferroni’s multiple comparisons test). **(F)** Distance moved in the arena during conditioning days for group-housed VTA-scrShank3 mice (*N* = 10) and VTA-shShank3 (*N* = 9) mice (two-way ANOVA: time × group interaction: *F*_(3,51)_ = 0.2209, *P* = 0.8814; group main effect: *F*_(1,17)_ = 3.388, *P* = 0.0832; main effect time: *F*_(3,51)_ = 3.707, *P* = 0.0173). **(G)** Representative occupancy heat-maps for group-housed VTA-scrShank3 mice at pre-TEST and **(H)** post-TEST. **(I)** Preference index calculated at pre- and post-TEST for group-housed VTA-scrShank3 mice (*N* = 10; paired *t*-test: *t*_(9)_ = 4.827; *P* = 0.0009). **(J)** Time spent in non-social and non-familiar conspecific-paired chamber for group-housed VTA-scrShank3 mice during pre- and post-TEST (*N* = 10; two-way RM ANOVA: time × chamber interaction: *F*_(1,9)_ = 25.35, *P* = 0.0007; main effect chamber: *F*_(1,9)_ = 1.918, *P* = 0.1994; main effect time: *F*_(1,9)_ = 0.4628, *P* = 0.5134; followed by Bonferroni’s multiple comparisons test). **(K)** Representative occupancy heat-maps for group-housed VTA-shShank3 mice at pre-TEST and **(L)** post-TEST. **(M)** Preference index calculated at pre- and post-TEST for group-housed VTA-shShank3 mice (*N* = 9; paired *t*-test: *t*_(8)_ = 2.103; *P* = 0.0687). **(N)** Time spent in non-social and non-familiar conspecific-paired chamber for group-housed VTA-shShank3 mice during pre- and post-TEST (*N* = 9; two-way RM ANOVA: time × chamber interaction: *F*_(1,8)_ = 4.943, *P* = 0.0329; main effect chamber: *F*_(1,8)_ = 2.651, *P* = 0.1027; main effect time: *F*_(1,8)_ = 0.119, *P* = 0.7322; followed by Bonferroni’s multiple comparisons test). **(O)** Learning index calculated for VTA-scrShank3 (*N* = 10) and VTA-shShank3 (*N* = 9; unpaired *t*-test: *t*_(17)_ = 0.3468; *P* = 0.7330). *N* indicates number of mice. Abbreviations: US, unconditioned stimulus; fr, fasciculus retroflexus; SNc, substantia nigra pars compacta; RN, red nucleus. *p < 0.05, ***p < 0.001.

**Figure 6:**
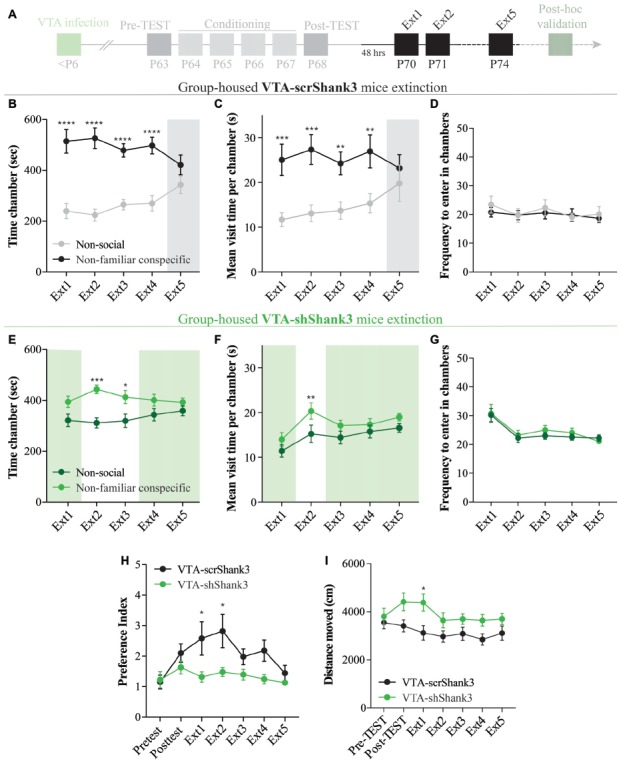
VTA SHANK3 downregulation alters the extinction of non-familiar conspecific-induced CPP. **(A)** Experimental time course of AAV-scrShank3/AAV-shShank3 VTA infection, CPP acquisition and extinction. **(B)** Time spent in non-social and non-familiar conspecific-paired chamber for group-housed VTA-scrShank3 mice across extinction sessions (*N* = 10; two-way RM ANOVA: time × chamber interaction: *F*_(4,36)_ = 4.624, *P* = 0.0041; main effect chamber: *F*_(1,9)_ = 18.32, *P* = 0.0020; main effect time: *F*_(4,36)_ = 1.126, *P* = 0.3595; followed by Bonferroni’s multiple comparisons test). **(C)** Mean visit time spent in non-social and non-familiar conspecific-paired chambers across extinction sessions for group-housed VTA-scrShank3 mice subjected to CPP (*N* = 10; two-way RM ANOVA: time × chamber interaction: *F*_(4,36)_ = 2.097, *P* = 0.1043; main effect chamber: *F*_(1,9)_ = 23.93, *P* = 0.0012; main effect time: *F*_(4,36)_ = 0.889, *P* = 0.4817; followed by Bonferroni’s multiple comparisons test). **(D)** Frequency to enter in non-social and non-familiar conspecific-paired chambers across extinction sessions for group-housed VTA-scrShank3 mice subjected to CPP (*N* = 10; two-way RM ANOVA: time × chamber interaction: *F*_(4,36)_ = 1.443, *P* = 0.2399; main effect chamber: *F*_(1,9)_ = 0.3101, *P* = 0.5912; main effect time: *F*_(4,36)_ = 0.5925, *P* = 0.6703). **(E)** Time spent in non-social and non-familiar conspecific-paired chambers for group-housed VTA-shShank3 mice during extinction (*N* = 9; two-way ANOVA: time × chamber interaction: *F*_(4,32)_ = 1.453, *P* = 0.2394; main effect chamber: *F*_(1,8)_ = 6.002, *P* = 0.0399; main effect time: *F*_(4,32)_ = 4.226, *P* = 0.0074; followed by Bonferroni’s multiple comparisons test).** (F)** Mean visit time spent in non-social and non-familiar conspecific-paired chambers across extinction sessions for group-housed VTA-shShank3 mice subjected to CPP (*N* = 9; two-way RM ANOVA: time × chamber interaction: *F*_(4,32)_ = 1.168, *P* = 0.3433; main effect chamber: *F*_(1,8)_ = 4.496, *P* = 0.0671; main effect time: *F*_(4,32)_ = 8.131, *P* = 0.0001; followed by Bonferroni’s multiple comparisons test). **(G)** Frequency to enter in non-social and non-familiar conspecific-paired chambers across extinction sessions for group-housed VTA-shShank3 mice subjected to CPP (*N* = 9; two-way RM ANOVA: time × chamber interaction: *F*_(4,32)_ = 1.708, *P* = 0.1725; main effect chamber: *F*_(1,8)_ = 0.6025, *P* = 0.4600; main effect time: *F*_(4,32)_ = 9.554, *P* < 0.0001). **(H)** Preference index calculated during pre-TEST, post-TEST and extinction sessions for group-housed VTA-scrShank3 mice (*N* = 10) and VTA-shShank3 (*N* = 9) mice (two-way ANOVA: time × group interaction: *F*_(6,102)_ = 2.118, *P* = 0.0575; group main effect: *F*_(1,17)_ = 5.436, *P* = 0.0323; main effect time: *F*_(6,102)_ = 4.953, *P* = 0.0002; followed by Bonferroni’s multiple comparisons test). **(I)** Distance moved in the arena during pre-TEST, post-TEST and extinction sessions for group-housed VTA-scrShank3 mice (*N* = 10) and VTA-shShank3 (*N* = 9) mice (two-way ANOVA: time × group interaction: *F*_(6,102)_ = 3.056, *P* = 0.0086; group main effect: *F*_(1,17)_ = 4.211, *P* = 0.0550; main effect time: *F*_(6,102)_ = 7.497, *P* < 0.0001; followed by Bonferroni’s multiple comparisons test). *N* indicates number of mice. Significance: *p < 0.05, **p < 0.01, ***p < 0.001, ****p < 0.0001.

### Statistical Analysis

Analysis of preference index, learning index and time in each chamber was conducted by performing Shapiro-Wilk analysis to assess the normality of sample distributions. *T*-test with Welch’s correction and paired *t*-test were used for comparisons between two sample groups when appropriate. When the normality was violated non-parametric Mann-Whitney and Wilcoxon rank-tests were applied. For multifactorial analysis, repeated-measures two-way ANOVA was performed, and *P* values of main effects and interaction were reported in the figure legends for each experiment. After significant main effects and interactions were revealed, Bonferroni *post hoc* tests were used for between/within group comparisons and reported in the figure legends or graphs. Significance level was set at *P* < 0.05. Statistical outliers were excluded when the time spent in either chamber during apparatus exploration (at pre-TEST, post-TEST or extinction sessions) was deviating for more than two standard deviations from the group mean. Within this manuscript, the non-familiar conspecific-induced CPP protocol was replicated four times in four different and independent animal batches.

## Results

### Non-familiar Conspecific-Induced CPP Assesses the Reinforcing Properties of Social Interaction

To study the role of SHANK3 in the VTA in mediating the reinforcing properties of social interaction, we modified previously published social-induced CPP protocols to obtain CPP mediated by interaction with a non-familiar conspecific in male mice. The paradigm started with a pre-TEST phase, during which mice explored for 15 min an empty apparatus consisting of two chambers characterized by different contextual cues (Figure 1A; see “Materials and Methods” section for further details). We then performed 4 days of conditioning, during which mie learnt to associate one compartment of the apparatus with the presence of a non-familiar (novel) social stimulus while the other compartment was left empty (non-social). During each conditioning day, we rapidly alternated for three times the exposure to the two chambers (Figure 1C). Twenty-four hours after the last conditioning session, we measured the acquisition of a place preference for one of the two chambers by quantifying the time spent exploring each compartment of the empty apparatus (Figure 1B). In addition, to compare the reinforcing properties of non-familiar vs. familiar conspecific, during the conditioning phase, experimental animals encountered in one chamber a non-familiar mouse, while in the other they were exposed to their familiar cage-mate (Figure 1D).

### The Acquisition of Non-familiar Conspecific-Induced CPP Does Not Require Single-Housing

Social CPP paradigms are often performed isolating the experimental mice before and/or during the conditioning. Single-housing, possibly through adaptations occurring within the dopaminergic system (Whitaker et al., 2013), enhances sociability (Panksepp and Beatty, 1980; Vanderschuren et al., 1995), increases the incentive value of social stimuli (Van den Berg et al., 1999; Trezza et al., 2009) and seems necessary to obtain social-induced CPP (Trezza et al., 2009). However, under these circumstances, it is difficult to determine whether the animals develop a CPP response because of the aversive properties of social isolation or because of the reinforcing properties of social interaction. Therefore, we tested whether housing conditions affected non-familiar conspecific-induced CPP acquisition in wildtype late adolescent mice (Figure 2A). By measuring the time spent in the non-familiar conspecific-paired chamber and the non-social chamber, we calculated a preference index (time spent in non-familiar conspecific-associated chamber/time spent in the non-social chamber) at the pre- and the post-TEST. We found that single-housed mice (during the conditioning) increased their preference index at the post-TEST compared to the pre-TEST (Figures 2B–D), indicating that they developed a preference for the non-familiar conspecific-associated chamber during conditioning. Additionally, at the post-TEST, single-housed mice spent a significantly longer time in the non-familiar conspecific-paired chamber compared to the non-social one (Figure 2E). This indicates that experimental animals were discriminating between contextual cues and expressing a preference for the ones associated with the interaction with a novel conspecific.

Subsequently, we subjected group-housed mice to the same conditioning paradigm used for single-housed animals. Surprisingly, we found that group-housed mice increased their preference for non-familiar conspecific-associated chamber (Figures 2F–H) and discriminated between non-familiar conspecific-associated vs. non-social chamber at the post-TEST (Figure 2I). Thus, the non-familiar conspecific-induced CPP paradigm, which relies on the direct and free exploration of a younger and non-familiar conspecific stimulus, allows us to study the reinforcing properties of social interaction without the need to single-house the experimental animals.

To prove the necessity of a contingency between contextual cues and social exposure for the acquisition of non-familiar conspecific-induced CPP, group-housed mice were exposed to the same number of social stimuli presentations, but in alternating chambers (contingency break) during the conditioning phase. In this case, we assigned the non-familiar conspecific-paired chamber as the first chamber in which they were exposed to the non-familiar social stimulus. Under these circumstances, the experimental animals did not increase their preference index (Figures 2J–L) and did not show any preference for one of the two chambers at the post-TEST (Figure 2M). Finally, to quantify the increase in place preference between pre- and post-TEST and to allow between-group comparisons, we calculated a learning index (preference index at the post-TEST/preference index at the pre-TEST). Confirming the validity of the non-familiar conspecific-induced CPP protocol, group-housed mice conditioned with the pairing protocol displayed a significantly higher learning index compared to animals subjected to the contingency break protocol (Figure 2N).

### The Interaction With a Juvenile, But Not Age-Matched, Non-familiar Conspecific Is Reinforcing

To understand whether the novelty component associated with non-familiar social stimuli is necessary to promote contextual learning, we assessed whether group-housed mice would develop any preference for either an age-matched familiar or a non-familiar juvenile mouse-paired context. By subjecting the animals to conditioning sessions in which they interacted with a non-familiar and a familiar stimulus in different chambers (Figures 1D, 3A), we observed an increase in the preference for the non-familiar conspecific-associated one (Figures 3B–D). Additionally, at the post-TEST, mice spent more time in the non-familiar conspecific-paired compartment compared to the one paired with a familiar mouse (Figure 3E). Finally, by comparing the learning index obtained from pairing protocols with either non-social session or familiar session, we found no differences in place preference acquisition (Figure 3F). Interestingly, when we subjected the animals to conditioning sessions in which they interacted with an age-matched non-familiar and a familiar stimulus in different chambers, we did not observe any preference (Figures 3G–K). Altogether, these results indicate that the social interaction with a non-familiar conspecific juvenile is reinforcing and that while the age of the stimulus plays an important role, the CPP is neither driven by social familiarity nor non-social pairing.

### Extinction of Non-familiar Conspecific-Induced CPP Is Affected by Housing the Condition

Considering that after the post-TEST, in adolescent rats, social-induced CPP responses are lost within three single exposures to the empty CPP apparatus (Trezza et al., 2009), we investigated whether the non-familiar conspecific-induced conditioned responses can also be extinguished. Thus, we exposed our experimental animals to six extinction sessions, as previously reported (Trezza et al., 2009). Starting 48 h after the post-TEST, both single-housed and group-housed mice freely explored the empty apparatus for 15 min once per day over 6 days (Figure 4A). During early extinction sessions, group-housed mice displayed longer exploration of the non-familiar conspecific-associated chamber as compared to the chamber where no social stimulus was present (Figure 4B). Moreover, group-housed mice showed an increased duration of the visit of non-familiar conspecific- compared to non-social compartment (Figure 4C), while no changes in the number of transitions in each chamber were observed (Figure 4D). At extinction session 6, group-housed mice lost their preference for the non-familiar-associated chamber (Figure 4B). However, single-housed mice spent a longer time in the non-familiar conspecific-associated compared to non-social compartment throughout all the extinction session performed (Figure 4E) and engaged in longer visits of non-familiar conspecific-associated chamber (Figures 4F,G). Thus, it is evident that at the sixth extinction session, while group-housed mice extinguished their preference for non-familiar conspecific-associated compartment, single-housed mice retained it (Figure 4H). Altogether, these results indicate that while housing conditions do not induce major changes in the acquisition of the non-familiar conspecific-induced CPP, single-housing affects the extinction of contextual associations induced by free interaction with a novel conspecific.

### VTA SHANK3 Downregulation Alters the Exploration of Non-familiar Conspecific Without Affecting CPP

Post-natal downregulation of the ASD-related protein SHANK3 in the VTA induces deficits in synaptic transmission, plasticity and social preference dynamics (Bariselli et al., 2016). Since both the excitatory transmission and plasticity at those inputs are essential for associative learning and extinction of reward-induced conditioned responses, we investigated the effects of VTA *Shank3* downregulation on the acquisition and extinction of non-familiar conspecific-induced CPP. More precisely, we downregulated *Shank3* in the VTA of neonatal mice (as previously described in Bariselli et al., 2016; Figure 5A), and we group-housed them after weaning for about 5 weeks. At 8 weeks of age (late adolescence), VTA-scrShank3 and VTA-shShank3 mice underwent non-familiar conspecific CPP acquisition and were subsequently sacrificed for histological validation (Figure 5B). By performing qPCR experiments on dissected midbrain regions, we found a pronounced downregulation of the longest SHANK3a transcript in AAV-shShank3 compared to AAV-scrShank3 infected animals (Figure 5C). This reduction was confirmed by normalizing the levels of the *Shank3a* mRNA to the mRNA encoding for the TH enzyme (Figure 5D), a marker of dopaminergic neurons (Stamatakis et al., 2013).

The downregulation of the ASD-associated protein Neuroligin 3 from VTA DA neurons induces an aberrant habituation to non-familiar conspecifics (Bariselli et al., 2018). To assess whether SHANK3 insufficiency at VTA neurons could induce a similar phenotype, we monitored both the distance moved and the reciprocal interaction of VTA-shShank3 and VTA-scrShank3 mice with their social stimuli. We observed that, while VTA-scrShank3 mice showed a progressive reduction in the interaction with the same social stimulus across conditioning days, VTA-shShank3 mice did not habituate to the social stimuli (Figure 5E). Importantly, these behavioral deficits were not associated with changes in the distance moved (Figure 5F).

When comparing pre- and post-TEST, VTA-scrShank3 mice increased their preference for the non-familiar conspecific-paired chamber (Figures 5G–I) and, at the Post-TEST, they displayed a longer exploration of the non-familiar conspecific-associated chamber (Figure 5J). When VTA-shShank3 mice were conditioned, they showed a trend towards a higher preference index between pre- and post-TEST (Figures 5K–M), and a significant preference for the non-familiar conspecific-associated chamber vs. the non-social chamber at the post-TEST (Figure 5N). Since the learning index of VTA-shShank3 mice was comparable to the one measured from VTA-scrShank3, we concluded that *Shank3* downregulation in the VTA was not inducing major abnormalities in the acquisition of non-familiar conspecific-induced CPP (Figure 5O).

### VTA SHANK3 Downregulation Alters the Extinction of Non-familiar Conspecific-Induced CPP

After 48 h, VTA-scrShank3 mice underwent an extinction protocol (Figure 6A) and, by the extinction session 5, they no longer displayed a preference for the non-familiar conspecific-paired chamber (Figure 6B). The extinction of the CPP at extinction 5 in VTA-scrShank3 mice was associated with a similar mean duration of the visits of non-familiar conspecific vs. non-social associated chamber (Figure 6C), with no differences in the number of transitions within the two compartments (Figure 6D). When VTA-shShank3 mice underwent extinction, we noticed that their preference for the non-familiar conspecific-paired chamber was not stable over time and already not significant by the fourth extinction session (Figure 6E). This effect was accompanied by a similar duration of the mean visit time of the two compartments at several extinction sessions (Figures 6F,G). To directly compare the extinction of non-familiar conspecific-induced place preference between VTA-scrShank3 and VTA-shShank3 mice, we compared the preference index across pre-TEST, post-TEST and extinction sessions. We found that VTA-shShank3, compared to VTA-scrShank3, had a significantly lower preference index, particularly during early extinction sessions indicating an accelerated extinction (Figure 6H). Importantly, VTA-shShank3 mice showed an increased distance moved during the experiment as compared to VTA-scrShank3 (Figure 6I).

## Discussion

Here, we tested whether the downregulation of the ASD-associated gene *Shank3* in the VTA is sufficient to alter the reinforcing properties of social interaction in mice and the extinction of the social-induced contextual associations. Reinforcing properties of social interactions have been previously tested by using protocols that require single-housing before and/or during conditioning. Here, by characterizing a modified version of previously published CPP paradigms, we show that while housing conditions (single-housing vs. group-housing) do not affect the acquisition of a preference for the compartment associated with a non-familiar conspecific, it influences the extinction of non-familiar conspecific-induced contextual associations. By using the newly characterized CPP protocol, we show that the downregulation of *Shank3* impairs habituation to non-familiar conspecifics and accelerates the extinction of social-induced contextual associations.

### Direct and Free Interaction With a Juvenile Non-familiar Conspecific Promotes Contextual Learning in Group-Housed C57Bl/6j Mice

Social novelty recognition induces territorial urinary marking (Arakawa et al., 2008), increases exploratory behavior (Ferguson et al., 2000), and produces exploratory preference, which is only expressed during the first 5 min of the three-chamber task (Nadler et al., 2004). For these reasons, we developed a CPP paradigm that requires short (5 min) and repeated episodes of free and direct interaction with a juvenile non-familiar sex-matched social stimulus (Bariselli et al., 2018). Contrary to what has been previously reported (Calcagnetti and Schechter, 1992; Thiel et al., 2008; Fritz et al., 2011; Kummer et al., 2011; Molas et al., 2017), we demonstrate that single-housing is not a necessary condition for the acquisition of a social-induced CPP. However, in the present study, we found that the direct interaction with a non-familiar juvenile mouse is necessary for social-induced contextual learning, which is not observed when experimental and stimuli subjects are age-matched. This difference could be the consequence of offensive behaviors. Considering that offensive behavior is a trait expressed by C57Bl/6 male mice (Crawley et al., 1997) and that aggression promotes place preference in CD1 male mice (Golden et al., 2016), further investigation is needed to address the role of antagonistic and offensive behavior in the acquisition of non-familiar conspecific-induced CPP. Additionally, considering the impact of sexual hormones on the acquisition of reward-induced place preference (Calipari et al., 2017), future studies will need to assess the acquisition of non-familiar conspecific-induced place preference in female mice at various stages of their estrous cycle.

### Single-Housing Affects the Extinction of Contextual Associations Induced by Non-familiar Interaction

Social-induced CPP undergoes extinction in adolescent rats (Trezza et al., 2009), and we demonstrated that social-induced conditioned responses could be induced and also lost in late adolescent mice. However, we found that housing conditions affect the extinction of the preference for the non-familiar conspecific-paired chamber. In single-housed compared to group-housed animals, the decreased rate of extinction of these conditioned responses might be due to the increased strength of the association between contextual cues (CS) and the presence of a non-familiar conspecific, which would strongly counteract the new association (CS-absence of social stimuli). The increased strength of the associations in single-housed mice could stem from the increased subjective value of social stimuli during the acquisition of the non-familiar conspecific-induced CPP as a consequence of the absence of social contacts in their home-cage. In fact, the saliency of social events is modulated by social context, as, compared to non-lonely individuals, lonely individuals convey higher attention and accuracy in decoding social cues (Pickett et al., 2004) and have a greater ability to recall socially-related information. Alternatively, the absence of social contacts in the home-cage during extinction might favor, in the presence of the contextual cues previously associated with social interaction availability, a behavioral strategy that maximizes the probability of engaging in social interactions (e.g., spending more time in the chamber paired with the exposure to a non-familiar conspecific). Also, in this case, the influence of the primary association on the behavioral outcome would be increased at the expense of the new CS-absence of social interaction association, thus resulting in a reduced extinction of the conditioned response. Considering the effects of single-housing on the extinction of non-familiar conspecific-induced CPP, investigating the behavioral and neuronal mal-adaptations induced by social isolation on the acquisition and extinction of social-induced conditioned responses might be beneficial for individuals affected by “loneliness” (*Cacioppo JT and Cacioppo S, The Lancet, “The growing problem of loneliness”*).

### *Shank3* Downregulation in the VTA Accelerates the Extinction of Non-familiar Conspecific-Induced CPP

In recent years, several genetic mouse models have been developed to study the involvement of each SHANK3 variant in synaptic and social behavior impairments (Bariselli and Bellone, 2016). The elimination of the major Shank3 isoforms induces aberrant social novelty recognition and social preference (Peça et al., 2011), the latter rescued by Shank3 re-expression at adulthood (Mei et al., 2016). Other mutants, with targeted mutation within the exon 4–9 sequence displayed sociability impairments (Wang et al., 2011) and altered male-female interaction (Bozdagi et al., 2010), while mice lacking Shank3 Pro-rich domain and exon 13 displayed altered social novelty recognition (Kouser et al., 2013; Jaramillo et al., 2017). Other behavioral traits, such as increased dominance has been reported in mice with point mutations of *Shank3* (Zhou et al., 2016). However, *Shank3* mutants lacking the exon 4–22 and exon 9, for example, displayed intact sociability (Wang et al., 2017; Bey et al., 2018). Moreover, considering the differential expression of *Shank3* across neuronal populations (Wang et al., 2014), specific behavioral tests are needed to assess the role of the protein in a discrete neuronal circuit. Previously, we showed that the developmental downregulation of *Shank3* in the VTA impaired social preference dynamics (Bariselli et al., 2016), pointing at a deficit in social motivation. Here, we found that insufficiency of SHANK3 in the VTA produces an aberrant habituation to non-familiar social stimuli, which is similar to our observation in another ASD-relevant animal model lacking Neuroligin 3 expression in VTA DA neurons (Bariselli et al., 2018). This indicates that different ASD-relevant mutations, within the same neuronal circuit, might lead to similar behavioral aberrancies.

Interestingly, we found that, although VTA-shShank3 mice discriminated between non-familiar conspecific-paired and non-social compartment at the post-TEST, they showed only a statistical trend towards an increase in their preference for the non-familiar conspecific-paired compartment before and after conditioning. These results might indicate subtle deficits in acquisition of non-familiar conspecific-induced contextual association. However, when VTA-shShank3 mice were repeatedly exposed to contextual cues in the absence of the social stimulus animal, their preference for the non-familiar conspecific-paired chamber became erratic and revealed an accelerated rate of extinction. This effect could be due to a reduced strength of the association as a consequence of *Shank3* downregulation in the VTA. According to the hypothesis that extinction is learning a new association, it is also plausible that in VTA-shShank3 mice the new association might easily overpower the weak primary association, thus resulting in an accelerated disappearance of the conditioned responses. To our knowledge, this is the first time that an extinction deficit is reported in an ASD-related genetic dysfunction restricted to the VTA, but further investigation is warranted to understand the behavioral and neurobiological mechanisms underlying aberrant learning and extinction of those associations.

In conclusion, the CPP is a behavioral procedure classically used to assess the reinforcing properties of drugs of abuse and natural rewards. The observation of social-induced CPP in group-housed mice demonstrates that direct social interaction with a non-familiar conspecific can be reinforcing and can be studied without the confounding behavioral and neurobiological mal-adaptations induced by social isolation. Extinction and reinstatement of drug-induced CPP responses are behavioral procedures used to assess “drug-seeking” behavior in mice (Itzhak and Martin, 2002). For this reason, many studies have focused their attention on the cellular, synaptic and circuit adaptations that underlie “drug-seeking” behavior to facilitate extinction, to reduce reinstatement (Thanos et al., 2009; Voigt et al., 2011; Malvaez et al., 2013; Prast et al., 2014) and, ultimately, to aid the research for treating addiction. Similarly, the methods of extinction, and possibly reinstatement, of non-familiar conspecific-induced CPP responses are a way to observe and assess “social-seeking” behavior in mice. This paradigm could prove instrumental in identifying mechanisms underlying “social-seeking,” and help to find pharmacological targets to promote social-induced learning in ASD patients.

## Author Contributions

SB, AC and ST performed the behavioral experiments. SB and AC prepared the figures. SM performed the qPCR analysis. SB and CB designed the study and wrote the manuscript.

## Conflict of Interest Statement

The authors declare that the research was conducted in the absence of any commercial or financial relationships that could be construed as a potential conflict of interest.
